# Analysis of the Differences in Volatile Organic Compounds in Different Rice Varieties Based on GC-IMS Technology Combined with Multivariate Statistical Modelling

**DOI:** 10.3390/molecules28227566

**Published:** 2023-11-13

**Authors:** Jin Chen, Ying Liu, Mi Yang, Xinmin Shi, Yuqin Mei, Juan Li, Chunqi Yang, Shihuang Pu, Jiancheng Wen

**Affiliations:** 1 Rice Research Institute, Yunnan Agricultural University, Kunming 650201, China; cj15126398640@163.com (J.C.); 15234827588@163.com (Y.L.);; 2 Lincang Seed Management Station, Lincang 677000, China

**Keywords:** rice, volatile components, gas chromatography–ion mobility spectrometry (GC-IMS), orthogonal partial least squares discriminant analysis, principal component analysis

## Abstract

In order to investigate the flavour characteristics of aromatic, glutinous, and nonaromatic rice, gas chromatography–ion mobility spectrometry (GC-IMS) was used to analyse the differences in volatile organic compounds (VOCs) amongst different rice varieties. The results showed that 103 signal peaks were detected in these rice varieties, and 91 volatile flavour substances were identified. Amongst them, 28 aldehydes (28.89~31.17%), 24 alcohols (34.85~40.52%), 14 ketones (12.26~14.74%), 12 esters (2.30~4.15%), 5 acids (7.80~10.85%), 3 furans (0.30~0.68%), 3 terpenes (0.34~0.64%), and 2 species of ethers (0.80~1.78%) were detected. SIMCA14.1 was used to perform principal component analysis (PCA) and orthogonal partial least squares discriminant analysis, and some potential character markers (VIP > 1) were further screened out of the 91 flavour substances identified based on the variable important projections, including ethanol, 1-hexanol, hexanal, heptanal, nonanal, (E)-2-heptenal, octanal, trans-2-octenal, pentanal, acetone, 6-methyl-5-hepten-2-one, ethyl acetate, propyl acetate, acetic acid, and dimethyl sulphide. Based on the established fingerprint information, combined with principal component analysis and orthogonal partial least squares discriminant analysis, different rice varieties were also effectively classified, and the results of this study provide data references for the improvement in aromatic rice varieties.

## 1. Introduction

Rice (*Oryza sativa* L.) is amongst the most important foods for humans and is irreplaceable. With rapid social and economic development and the continuous improvement in people’s lives, the quality [1] and edibility [2] of rice have received increasing attention in China and abroad. Aroma is an important quality indicator of rice and has a direct impact on consumer desires to buy rice. Aromatic rice contains unique volatile aroma substances and is rich in nutrients, such as amino acids, and trace elements and is favoured by consumers and the market [3,4]. The study of rice aroma traits and their aroma components has long been an important direction of research on rice-quality research. To date, hundreds of volatile substances have been detected in rice [5], including aldehydes, ketones, esters, alcohols, heterocycles, and alkenes [6]. The aroma of rice is generated via the comprehensive action of various aromatic volatile components. The differences in the types and proportions of volatile substances lead to differences in rice aroma [7,8]. Therefore, the in-depth study of volatile components in different varieties of aromatic rice is of great significance for the improvement in aromatic rice varieties and the development of high-end, high-quality rice.

With the rapid development of technology, which detects volatile substances, detailed sensory analysis of rice odour quality can be performed, and volatile substances can be quantitatively and qualitatively identified. In recent years, gas chromatography–ion mobility spectrometry (GC-IMS) technology has been used for the gas-phase separation and detection of volatile compounds, and the separation and detection of chemical ionic substances are performed through variations in the electric field mobility of gas-phase ions [9]. This technique combines the outstanding separation characteristics of gas chromatography with the advantages of ion mobility spectrometry, which include its fast response, high sensitivity, low cost, and lack of sample pretreatment in contrast to other detection techniques [10]; as a result, the method has become one of the emerging technologies used to analyse and detect volatile flavour substances. Moreover, the combination of multivariate statistics, such as principal component analysis and orthogonal partial least squares discriminant analysis, can achieve the screening of different samples for different flavours [11,12,13].

A large amount of aromatic rice resources is available in Yunnan, China. In the 1980s, the aromatic rice variety Diantun 502 was bred [14]; its aroma traits are mainly controlled by the aroma gene *badh2* [15], and the rice has a rich aroma and good taste [16]. Diantun 502 is the main variety used to produce aromatic and soft high-quality rice in Yunnan Province. This variety has become an important basic material for aromatic rice breeding in Yunnan. After practices were performed to improve rice, it was found that newly improved varieties showed significant differences in aroma intensity, and there may be some important aroma components that are still unknown. There have been many reports on the study of volatile substances in aromatic rice and nonaromatic rice [17,18]. However, studies comparing the differences in rice volatile compounds amongst aromatic rice, nonaromatic rice, and aromatic glutinous rice varieties are still rarely reported. Therefore, the purpose of this study was to use GC-IMS combined with multivariate statistical analysis (PCA and OPLS-DA) to find some potential markers to resolve the phenomenon of the significant differences in aroma amongst the improved progeny of aromatic rice Diantun 502, and these data can also provide a theoretical basis for the breeding of aromatic rice in Yunnan. It was also verified that GC-IMS could effectively distinguish aromatic rice, nonaromatic rice, and aromatic glutinous rice varieties.

## 2. Results and Discussion

### 2.1. Direct Comparison of the Differences in Volatile Compounds Amongst Different Rice Varieties

**Figure 1:**
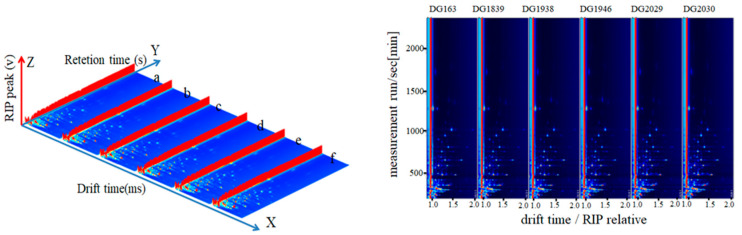
(**left**,**right**) GC-IMS 3D spectra (a~f: DG163, DG1839, DG1938, DG1946, DG2029, DG2030) and GC-IMS 2D spectra of volatile substances in different rice varieties.

**Figure 2:**
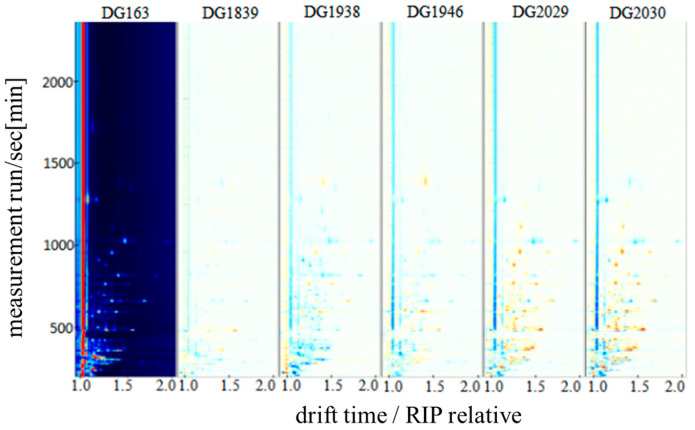
GC-IMS two-dimensional spectra of volatile substances in different rice varieties (difference comparison map).

According to the retention time, migration time, and peak intensity, Reporter software was used to analyse the three-dimensional spectrum (left in Figure 1) and the two-dimensional spectrum (right in Figure 1). In Figure 1 (left), the ion migration spectra are compared via 3D spatial distribution to obtain the 3D spectra (left in Figure 1; *X*, *Y*, and *Z* axes represent drift time, retention time, and peak intensity, respectively), but due to the inconvenience of observation, the top view (right in Figure 1 and Figure 2) was selected, at the same time, to compare the differences. In Figure 1 (right), on both sides of the RIP peak, each dot represents a volatile substance, and the colour represents the concentration of the substance: white represents a low concentration, red represents a high concentration, and darker colours represent higher concentration. The GC-IMS 3D atlas was observed from the outside (Figure 1, left), and it was relatively difficult to visually compare the volatile components of six different rice varieties; thus, the GC-IMS 2D spectrum was obtained by projecting the 3D spectrum (Figure 1, right).

To visually compare the differences in the volatile flavour substances of different rice varieties, difference comparison spectra of the volatile substances of six different rice varieties were obtained by subtracting the DG163 spectrum, as shown in Figure 2. The dark-blue area indicates a low concentration of the substance, and the dark-red area indicates a high concentration of the substance. The results show that the aromatic glutinous rice varieties DG2030 and DG2029 were the most abundant in volatile substances, followed by the aromatic rice varieties DG163, DG1839 and DG1946, and the nonaromatic rice variety DG1938 had the lowest concentration. Different rice volatiles were better separated by the GC-IMS features, so there were relative differences in the GC-IMS feature spectra of different rice varieties, suggesting that the content of volatile flavouring substances varied from one rice to another (red dashed box area in Figure 2). This may be related to the differences in factors, such as raw material varieties and different nutrient chemical compositions [19,20].

### 2.2. Qualitative Analysis of GC-IMS Spectra of Different Rice Varieties

**Figure 3:**
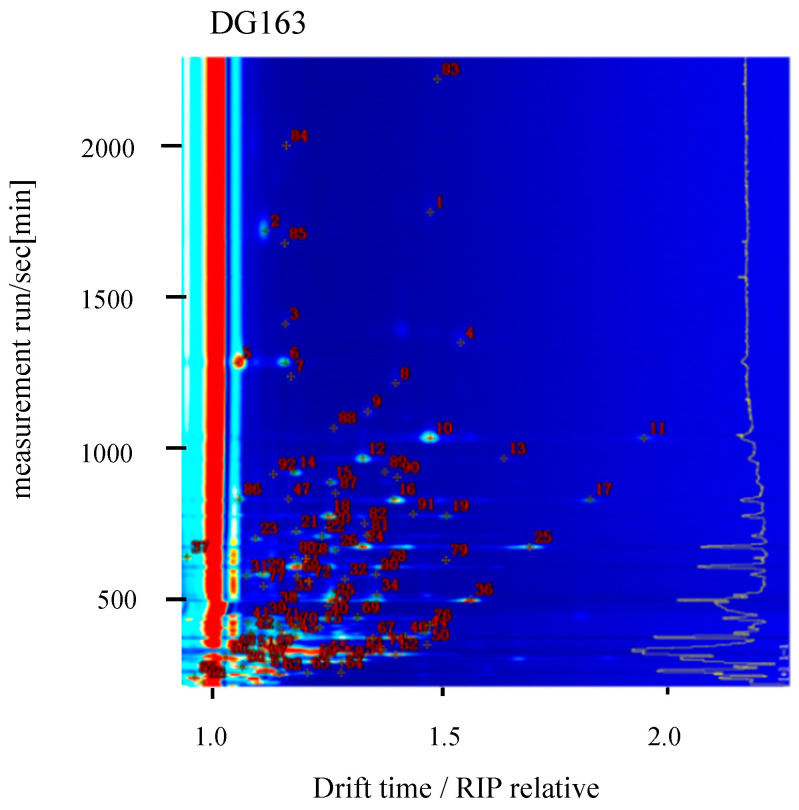
Qualitative analysis spectrum of volatile substances in rice variety DG163 via GC-IMS.

The retention time and migration time of volatile flavour compounds from different varieties of rice were compared, and the n-ketone C4~C9 calibration solution was used as an external standard reference to calculate the retention indices of the volatile compounds. The NIST built-in Library Search in GC-IMS was used to calculate the retention index. The database was matched with the IMS migration time database to qualitatively analyse the volatile substances [21] and perform quantitative analysis based on the signal peak intensity [22]. Figure 3 shows the qualitative analysis chromatogram obtained for the variety DG163. The qualitative and quantitative analyses of the volatile compounds from the six rice varieties are shown in Table 1. A total of 91 volatile substances were identified amongst 103 signal peaks, including 28 aldehydes, 24 alcohols, 24 ketones, 14 ketones, 12 esters, 5 acids, 3 furans, 3 terpenes, 2 ethers, 1 other, and 11 unknown components. The volatile components are mainly compounds such as aldehydes, alcohols, esters, and ketones [6,23]. Aldehydes include (E)-2-octenal, nonanal, octanal, heptanal, hexanal, and valeraldehyde, which are mainly derived from lipid oxidation [24] and generally have a pleasant, fruity, floral, and delicate aroma (for example, (E)-2-octenal generates a green aroma, nonanal generates a fatty aroma, and octanal generates a citrus aroma; these are the main contributors to rice aroma with a low threshold [25]). Alcohols include 1-octen-3-ol, 1-hexanol, 1-pentanol, etc., which generally originate from the oxidation of fat and have an aromatic, vegetal, rancid, and earthy odour and are an important component of rice aroma [26]. Ketones include 2-heptanone, acetone, 1-penten-3-one, etc., which are produced by the oxidation of unsaturated fatty acids, the Maillard reaction, etc., and have pleasant odours, such as fresh, creamy, and fruity aromas, and the aroma threshold is relatively low [27]. Esters include propyl acetate, ethyl acetate, and ethyl formate, which have fruity, sweet, and wine-like aromas and endow rice with a light fruity aroma. Esters generally do not make a significant contribution to the development of the overall aroma but rather play a role in accentuating the aroma [28]. Furans include 2-pentylfuran and 2-ethylfuran, which exhibit beany, fruity, and earthy aromas, amongst others [29]. Acids include acetic acid and butyric acid, which are generally produced via the hydrolysis of triglycerides and phospholipids or the oxidation of alcohols and aldehydes. Acetic acid is a low-level saturated acid with a strong pungent odour [30], which is undesirable. Other compounds, such as alkanes, alkenes, terpenes, and ethers, are common in all varieties and are generally considered not to contribute to rice aroma.

### 2.3. Comparison of GC-IMS Fingerprints of Different Rice Varieties

**Figure 4:**
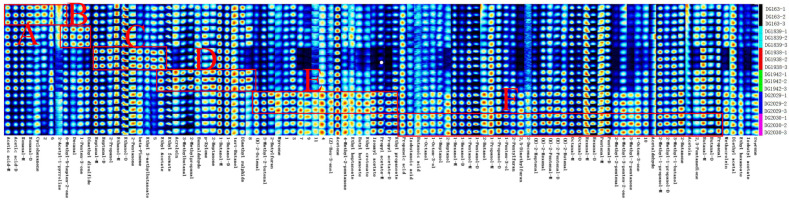
Fingerprints of volatile components in different rice varieties. Note: rows represent all signal peaks selected in the samples, and columns represent signal peaks of volatile components in different samples.

All peaks were selected to compare the fingerprints and differences in the flavour substances of the different rice varieties (Figure 4). Each row in the figure represents all signal peaks selected in one rice sample, and each column represents the signal peaks of the same volatile organic compound in different rice samples. Due to the higher concentration of some volatile compounds, they will exhibit different forms, such as monomers (Ms) and dimers (Ds), and corresponding migration peaks will appear. The data of all identified flavour substances were selected. The fingerprints were generated via the built-in plug-in of the instrument. GC-IMS fingerprints showed that the contents of major volatile components amongst the six improved rice lines were significantly different, with obvious characteristic peak areas. Relatively high contents of acetic acid, nonanal, and cyclohexanone are in the aromatic rice DG163 (region A). The aromatic rice DG1839 contains relatively high levels of 2-acetyl-1-pyrroline, 6-methyl-5-hepten-2-one, decanal, 1-penten-3-one, and dimethyl disulphide (region B). Relatively high levels of heptanal, isopropanol, 2-pentanone, β-pinene, ethyl isovalerate, and ethyl acetate are in the nonaromatic rice DG1938 (region C). The contents of acrolein, 3-methylbutanal, isobutanol, benzaldehyde, p-xylene, 2-heptanone, 1-butanol dimer, tert-butanol, and dimethyl sulphide in the aromatic rice DG1946 are relatively high (region D). In contrast, most of the volatile components are high in the aromatic glutinous rice varieties DG2029 and DG2030, with relatively high contents of 2-butylfuran, laurin, butyl butyrate, and butenoic acid ethyl ester in DG2029 (region E), and the contents of isobutyric acid, butyric acid, 1-penten-3-ol, 2-pentylfuran, 2,5-dimethylfuran, (E)-2-hexenal, (E)-2-butene aldehyde, 2-methylpropanol-M, 2-methylpropanol-D are relatively high (region F). From a macroscopic point of view, the contents of the main volatile compounds are different amongst aromatic rice, aromatic glutinous rice, and nonaromatic rice. The relative contents of volatile components of the two aromatic glutinous rice varieties are all higher, followed by the three aromatic rice varieties, and the nonaromatic rice possesses the lowest content.

**Figure 5:**
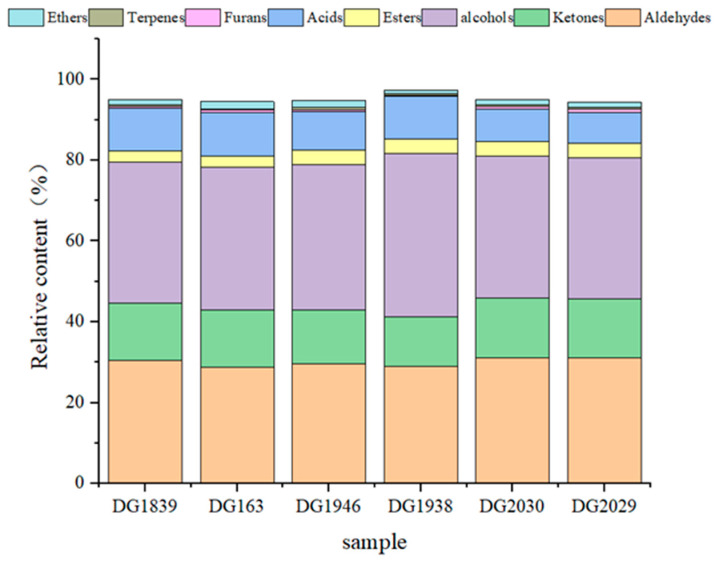
Relative contents of volatile substances in different rice varieties.

To clearly present the differences in rice flavour substances between different varieties, the relative contents of flavour substances in the varieties were obtained by normalizing the peak volumes of each flavour substance on the fingerprints (Figure 5). The flavour substances of the different rice varieties contained alcohols, aldehydes, ketones, acids, and esters, in which the relative contents of alcohols, aldehydes and ketones were higher. The relative contents of aldehydes in DG1839, DG163, DG1946, DG1938, DG2030, and DG2029 were 30.38%, 28.89%, 29.65%, 28.94%, 31.17%, and 31.04%, respectively; those of alcohols were 34.97%, 35.18%, 35.95%, 40.52%, 35.17%, and 34.85%, respectively; those of ketones were 14.23%, 14.17%, 13.28%, 12.26%, 14.74%, and 14.67%, respectively; those of acids were 10.52%, 10.85%, 9.62%, 10.52%, 8.08%, and 7.8%, respectively; those of esters were 2.83%, 2.82%, 3.55%, 2.3%, 3.87%, and 4.15%, respectively; those of ethers were 1.32%, 1.78%, 1.64%, 0.8%, 1.32%, and 1.23%, respectively; those of furans were 0.4%, 0.44%, 0.42%, 0.3%, 0.62%, and 0.68%, respectively; and those of terpenes were 0.35%, 0.34%, 0.64%, 0.46%, 0.45%, and 0.55%, respectively. Lin et al. [31] used the SPME/GC-MS technique to determine the volatile components of rice grains, which were mainly alcohols, aldehydes, ketones, esters, hydrocarbons, organic acids, and heterocyclic compounds, with hydrocarbons being the most abundant, followed by aldehydes and ketones. In contrast, studies by Sun [32], Bian [33], and Zhu et al. [34] used the GC-IMS technique to detect volatiles from different rice varieties and detected the highest number of aldehyde species followed by alcohols and ketones, which is consistent with the results of this study. The reason for these differences may be the variation in the materials and the detection techniques. Some researchers compared the results of GC-IMS and GC-MS analyses and found differences. The volatile components determined using the GC-IMS method were more informative in terms of characteristic peaks. It was hypothesised that the main reason for this was due to the different pretreatment methods. When volatile compounds are determined via the GC-MS method, water vapour distillation of the samples is required, whereas no pretreatment is needed for the samples determined using the HS-GC-IMS method. The direct determination of samples after crushing maximises the retention of volatile components in the samples and shows some advantages in the identification of characteristic components [35,36,37].

### 2.4. Principal Component and Orthogonal Least Squares Discriminant Analyses of the Volatile Components of Different Rice Varieties

**Figure 6:**
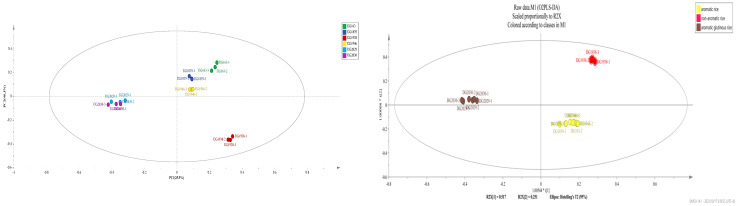
PCA diagram (**left**) and OPLS-DA model (**right**) of volatile substances of different rice varieties.

Principal component analysis was performed on the relative contents of volatile substances in different rice varieties (Figure 6, left). The contribution rate of PC1 was 25.9%, the contribution rate of PC2 was 46.3%, and the cumulative contribution rate was 72.2%. As a result of PCA, the aromatic, nonaromatic, and aromatic glutinous rice varieties were clustered; three aromatic rice varieties (DG163, DG1839, and DG1946) were clustered; two aromatic glutinous rice varieties (DG2029 and DG2030) were clustered; and a nonaromatic rice variety (DG1938) was clustered alone.

**Figure 7:**
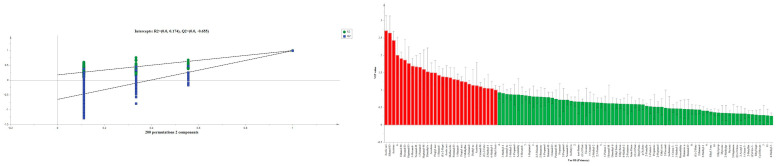
OPLS-DA model validation and VIP values of different rice varieties (Compounds highlighted in red are potential signature markers for VIP > 1).

OPLS-DA was performed on the volatile components of the different rice samples (Figure 6, right). OPLS-DA could effectively differentiate aromatic rice, nonaromatic rice, and aromatic glutinous rice. Three aromatic rice varieties (DG163, DG1839, and DG164) were distributed in the fourth quadrant; two aromatic glutinous rice varieties (DG2029 and DG2030) were distributed in the second quadrant. A nonaromatic variety DG1938 was distributed in the first quadrant. The model is usually evaluated using the independent variable fit index R^2^_X_ (cum), the dependent variable fit index R^2^_Y_ (cum), and the model prediction index Q^2^ (cum). R^2^_X_ and R^2^_Y_ denote the explanatory rate of the constructed model for the X and Y matrices, respectively, and Q^2^ denotes the predictive power of the model. Q^2^ and R^2^ close to 1 imply a better model fit and higher than 0.4 indicates that the model is acceptable, while a Q^2^ larger than 0.5 indicates that the model has good predictive power [38,39]. The model R^2^_X_ = 0.95, R^2^_Y_ = 0.994, and Q^2^ = 0.99, which are all close to 1, indicate that the model fits well, has good predictive ability, and is reliable for analysing signature compounds [40]. In order to avoid the overfitting phenomenon, the OPLS-DA model was cross-validated (200 permutation fits). The regression slopes of R^2^ and Q^2^ were both >0, and the intercepts were <0.5 and −0.5, respectively, indicating that the model did not overfit (left in Figure 7), was stable, and had good predictive ability.

Variable importance projection values greater than 1 (VIP value > 1) generally indicate key flavour substances. Amongst the 6 different rice varieties, there were 30 volatile substances with VIP values > 1 (including dimers of some substances), including acetic acid, ethanol, acetone,6, hexanal, heptanal, 1-hexanol, nonanal, 1-pentanol, heptanal, dimethyl sulphide, acrolein, propyl acetate, 1-butanol, (E)-2-heptenal, methacrolein, valeraldehyde, 3-methylbutyraldehyde, acetal, acetoin, octenal, trans-2-octenal, 2-methyl-1-propanol, 6-methyl-5-hepten-2-one, and ethyl acetate. In this study, the contents of each key flavour compound were significantly different in aromatic (DG163, DG1839, and DG1946), nonaromatic (DG1938), and aromatic glutinous rice (DG2029, DG2030) varieties (Table 1).

Amongst the key flavour substances of aldehydes, the content of octanal (M + D) reached significantly different levels for aromatic glutinous rice, aromatic rice, and nonaromatic rice. The studies of Yang and Sarika et al. also showed that the octanal content of aromatic rice was significantly higher than that of nonaromatic rice [17,41], which is consistent with the results of this study; the hexanal content (M + D) in aromatic glutinous rice was significantly higher than that in aromatic and nonaromatic rice; hexanal is a lipid oxidation marker during rice ageing [42]; and it has been shown that amylose can react with hexanal to form a V-type crystal complex [43]. However, aromatic glutinous rice has a very low content of amylose, which may be due to the fact that glutinous rice produces fewer crystal complexes, resulting in higher levels of detected hexanal. In terms of alcohols, the content of 1-hexanol (M + D) in aromatic glutinous rice was significantly higher than that in nonaromatic and aromatic rice. An analysis by Peng et al. [44] showed that nonaromatic rice had a higher content of 1-hexanol than glutinous rice, which is inconsistent with the present study and may be due to the difference in materials, as well as detection techniques. Amongst ketones, the contents of acetone and 6-methyl-5-hepten-2-one in aromatic rice and aromatic glutinous rice were significantly higher than those in nonaromatic rice. In addition, the characteristic rice aroma compound 2-acetyl-1-pyrroline was detected, and the 2-AP content in aromatic rice was significantly higher than in nonaromatic rice and aromatic glutinous rice. Most studies believe that the aromatic substance 2-AP is caused via the mutation of the gene encoding betaine dehydrogenase *Badh2*, the loss of betaine dehydrogenase activity, and the production of nonfunctional BADH2 protein, which results in the accumulation of 2-AP [45,46]. It has also been reported that the 2-AP content of varieties with high amylose content is considered to be lower than that of varieties with low amylose content (glutinous rice) because 2-AP interacts with amylose to form a V-type complex [43], which may be a reason why the 2-AP content of the nonaromatic rice DG1938 and the aromatic glutinous rice DG2029 and DG2030 did not have any significant difference in the present study, but the appearance of the 2-AP feature profile of the nonaromatic rice DG1938 was much darker in fingerprints.

In the present study, a small amount of 2-AP was also detected in nonaromatic rice DG1938, which showed that both aromatic and nonaromatic rice produce the characteristic aromatic compound 2-AP, with the only difference being the concentration between them. However, since some nonaromatic rice contains higher levels of linoleic acid, linolenic acid, and lipid oxidase, more secondary oxidation products are formed, so this could be an important reason why both aromatic and nonaromatic rice, despite both containing 2-AP, smell less aromatic than the flavoured rice [8]. Therefore, the PCA and OPLS-DA analyses based on GC-IMS data all showed significant differences in key flavour substances amongst aromatic rice, nonaromatic rice and aromatic glutinous rice. The results also showed that it was feasible to apply GC-IMS to detect and identify substances that produce flavours in rice.

## 3. Materials and Methods

### 3.1. Experimental Materials

**Figure 8:**
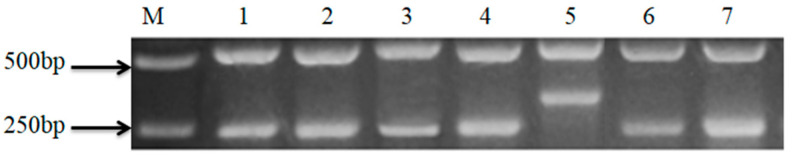
PCR analysis of aromatic gene *badh2* locus of aromatic rice Diantun502 and its improved lines. Note: M: DNA ladder marker; 1: DT502; 2: DG163; 3: DG1839; 4: DG1946; 5: DG1938; 6: DG2029; 7: DG2030.

As shown in Table 2, six rice varieties were used in the test, in which Diangu 163, Diangu 1839, and Diangu 1946 were aromatic rice; Diangu 1938 was nonaromatic rice; and Diangu 2029 and Diangu 2030 were aromatic glutinous rice with aroma traits and waxy properties. Further molecular identification of the aroma gene *badh2* locus showed that (Figure 8), five lines with the aroma trait appeared with two bands of about 580 bp and 255 bp in size, which were consistent with the band pattern of DT502, and were recessive pure genotypes *badh2/badh2*, which were aroma rice; one line was without the aroma trait, which appeared with two bands of about 580 bp and 355 bp in size, which were inconsistent with the band pattern of DT502, and were dominant pure genotypes *badh2/badh2*, which were nonaromatic rice. All materials were provided by the Rice Research Institute of Yunnan Agricultural University.

### 3.2. Flavour-Gene-Specific Primer Analysis

Referring to Bradbury [45], specific primers were used to analyse the *badh2* locus of the scent gene. The primer sequences were synthesised by Shanghai Sangong Bioengineering Technology Service Co Ltd. (Shanghai, China) (Table 3). The PCR reaction system was 15 μL, including 7.5 μL mix, 1 μL DNA, 4.5 μlddH_2_O, and 0.5 μL of each of the four primers. The reaction procedure was as follows: denaturation at 94 °C for 5 min, denaturation at 94 °C for 30 s, annealing at 54 °C for 30 s, and extension at 72 °C for 1 min, with a total of 29 cycles and extension at 72 °C for 10 min.

### 3.3. Instruments and Equipment

The FlavourSpec^®^ sensitive analyser (G.A.S, Rehden, Germany) and a quality control system (GC-IMS system equipped with a CTC automatic headspace sampler, Laboratory Analytical Viewer (LAV) analysis software, and Library Search qualitative software) were used.

### 3.4. Methods

#### 3.4.1. Sample Preparation

The method of Song [47] was used to process samples. Different varieties of rice were collected, dried in the air, browned, and refined; 2.0 g of refined rice was weighed, incubated at 60 °C for 15 min, and placed in a 20 mL headspace flask; and each sample was assayed in parallel three times.

#### 3.4.2. HS-GC-IMS Analysis

Autosampling conditions were as follows: incubation temperature, 60 °C; incubation time, 15 min; injection mode, headspace injection; heating mode, oscillatory heating; oscillation rate, 500 r/min; injection needle temperature, 85 °C; injection volume, 500 uL without shunt mode.

GC conditions were as follows: column type: MXT-WAX capillary column (30 m × 0.53 mm × 1 µm, RESTEK, Bellefonte, PA, USA); column length: 30 m; inner diameter: 0.53 mm; film thickness: 1.0 µm; column temperature: 60 °C; running time: 20 min; carrier gas: high-purity nitrogen; carrier gas flow rate: 2 mL/min for 0–2 min, 10 min to 10 mL/min, 20 min to 10 mL/min, 10 mL/min at 0–2 min, rising to 100 mL/min at 10 min; running time: 20 min.

IMS conditions were as follows: drift tube temperature, 45 °C; drift gas, N2 (purity ≥99.999%); drift gas flow rate, 150 mL/min; radiation source, β-rays (tritium, 3H); ionisation mode, positive ion.

### 3.5. Data Processing

On the FlavourSpec^®^ sensitive analyser, the Vocal software provided with the instrument was used for information acquisition and analysis. The Reporter and Gallery Plot plug-ins are built into the application software to plot the 2D spectrum and fingerprint of the volatile components of the sample. The n-ketone C4~C9 calibration solutions (2-butanone, 2-pentanone, 2-hexanone, 2-nonanone, 2-heptanone, and 2-octanone) were used as external standard reference substances to calculate the retention indices of the volatile components. They were matched with the National Institute of Standards and Technology (NIST) and IMS databases built into the application to perform qualitative analysis on the volatile components, the relative content of each volatile component was calculated via the peak area normalization method, and three samples were analysed in parallel. The analysis of significant differences was performed using SPSS 26.0 software. Three repetitions were performed; data were expressed as mean ± SD and analysed for significance using Duncan’s test (*p* < 0.05); and PCA and orthogonal partial least squares discriminant analysis (OPLS-DA) were completed using SIMCA 14.1 software.

## 4. Conclusions

In this study, the GC-IMS technique was used to analyse volatile compounds from six different sources of aromatic rice, aromatic glutinous rice, and nonaromatic rice. A total of 103 volatile components were detected, including 28 aldehydes, 24 alcohols, 14 ketones, 12 esters, 5 acids, 3 furans, 3 terpenes, 2 ethers, 1 other compound, and 11 unknown components. A model with good stability and predictability was established using OPLS-DA. A total of 30 potential markers (VIP > 1) were screened from 103 volatile substances, including ethanol, 1-hexanol, 1-butanol, hexanal, heptanal, nonanal, (E)-2-heptenal, octanal, trans-2-octenal, pentanal, acetone, 6-methyl-5-hepten-2-one, ethyl acetate, propyl acetate, acetic acid, and dimethyl sulphide. This study established a visualization atlas of the different flavour substances of the various aromatic rice, nonaromatic rice, and aromatic glutinous rice varieties, as well as clarified the differences in the main flavour substances amongst different aromatic rice, nonaromatic rice, and aromatic glutinous rice samples. However, the number of selected varieties is limited, and the source is single, so the number of samples still needs to be increased to establish volatile fingerprints for different types of aromatic rice, nonaromatic rice, and aromatic glutinous rice, which can provide a data reference for flavour substance analysis and flavour identification of different types of rice.

## Figures and Tables

**Table 1:** Qualitative results of volatile substances in six different varieties of rice.

No	Compounds	Retention Index (RI)	Retention Times (s)	Drift Times (ms)	Relative Content (%)					
					DG163	DG1839	DG1938	DG1946	DG2029	DG2030
1	1-Octanol	1655.8	1782.45	1.47798	0.0066 ± 0.0006 ^abc^	0.0065 ± 0.0001 ^bc^	0.0055 ± 0.0003 ^d^	0.0057 ± 0.0005 ^cd^	0.0075 ± 0.0007 ^a^	0.007 ± 0.0006 ^ab^
2	Propanoic acid	1639.9	1721.92	1.11696	0.0130 ± 0.0019 ^a^	0.0144 ± 0.0008 ^a^	0.0148 ± 0.0007 ^a^	0.0149 ± 0.0006 ^a^	0.0137 ± 0.0010 ^a^	0.0139 ± 0.0006 ^a^
3	Benzaldehyde	1547.8	1409.97	1.15925	0.0031 ± 0.0003 ^ab^	0.0030 ± 0.0003 ^b^	0.0035 ± 0.0001 ^a^	0.0034 ± 0.0002 ^a^	0.0028 ± 0.0003 ^bc^	0.0024 ± 0.0001 ^c^
4	Decanal	1526.8	1347.12	1.54628	0.0042 ± 0.0002 ^b^	0.0048 ± 0.0000 ^a^	0.0035 ± 0.0001 ^cd^	0.0038 ± 0.0001 ^c^	0.0025 ± 0.0002 ^e^	0.0033 ± 0.0004 ^d^
5	Acetic acid-M	1503.1	1279.61	1.05517	0.0742 ± 0.0017 ^a^	0.0706 ± 0.0010 ^b^	0.0695 ± 0.0012 ^b^	0.0641 ± 0.0016 ^c^	0.0496 ± 0.0024 ^d^	0.0491 ± 0.0010 ^d^
6	Acetic acid-D	1503.1	1279.61	1.15925	0.0126 ± 0.0014 ^a^	0.0127 ± 0.0002 ^a^	0.0111 ± 0.0006 ^b^	0.0111 ± 0.0008 ^b^	0.0067 ± 0.0009 ^c^	0.0066 ± 0.0002 ^c^
7	1-Octen-3-ol	1486	1233.05	1.17226	0.0017 ± 0.0003 ^bc^	0.0016 ± 0.0002 ^c^	0.0016 ± 0.0000 ^c^	0.0017 ± 0.0001 ^bc^	0.0019 ± 0.0001 ^ab^	0.0021 ± 0.0000 ^a^
8	1-Heptanol	1477.2	1209.77	1.40318	0.0050 ± 0.0003 ^cd^	0.0056 ± 0.0001 ^c^	0.0041 ± 0.0005 ^e^	0.0048 ± 0.0003 ^d^	0.0070 ± 0.0003 ^a^	0.0063 ± 0.0005 ^b^
9	(E)-2-Octenal	1440.3	1116.65	1.34138	0.0033 ± 0.0003 ^d^	0.0030 ± 0.0001 ^d^	0.0032 ± 0.0001 ^d^	0.0037 ± 0.0001 ^c^	0.0068 ± 0.0001 ^a^	0.0062 ± 0.0001 ^b^
10	Nonanal-M	1402.3	1028.19	1.47798	0.0293 ± 0.0004 ^a^	0.0283 ± 0.0004 ^a^	0.0255 ± 0.0006 ^b^	0.0260 ± 0.0019 ^b^	0.0191 ± 0.0008 ^c^	0.0207 ± 0.0002 ^c^
11	Nonanal-D	1402.3	1028.19	1.94958	0.0039 ± 0.0002 ^a^	0.0034 ± 0.0003 ^b^	0.0027 ± 0.0003 ^c^	0.0029 ± 0.0004 ^c^	0.0018 ± 0.0002 ^d^	0.0020 ± 0.0001 ^d^
12	1-Hexanol-M	1370.8	960.16	1.33012	0.0153 ± 0.0008 ^e^	0.0175 ± 0.0002 ^d^	0.0226 ± 0.0002 ^b^	0.0184 ± 0.0001 ^c^	0.0230 ± 0.0001 ^b^	0.0241 ± 0.0007 ^a^
13	1-Hexanol-D	1370.2	958.94	1.64097	0.0022 ± 0.0001 ^e^	0.0026 ± 0.0002 ^d^	0.0035 ± 0.0001 ^cd^	0.0028 ± 0.0001 ^d^	0.0042 ± 0.0001 ^b^	0.0048 ± 0.0003 ^a^
14	6-Methyl-5-hepten-2-one	1348	913.84	1.17965	0.0082 ± 0.0002 ^a^	0.0082 ± 0.0002 ^a^	0.0052 ± 0.0001 ^d^	0.0067 ± 0.0001 ^b^	0.0058 ± 0.0001 ^c^	0.0051 ± 0.0001 ^d^
15	(E)-2-Heptenal	1331.1	880.92	1.25884	0.0059 ± 0.0002 ^c^	0.0052 ± 0.0001 ^d^	0.0040 ± 0.0000 ^e^	0.0070 ± 0.0001 ^b^	0.0108 ± 0.0003 ^a^	0.0107 ± 0.0002 ^a^
16	Octanal-M	1299.4	822.41	1.39744	0.0173 ± 0.0007 ^c^	0.0187 ± 0.0002 ^b^	0.0148 ± 0.0002 ^d^	0.0169 ± 0.0004 ^c^	0.0190 ± 0.0001 ^ab^	0.0195 ± 0.0003 ^a^
17	Octanal-D	1299.4	822.41	1.83104	0.0044 ± 0.0002 ^c^	0.0045 ± 0.0002 ^bc^	0.0028 ± 0.0002 ^e^	0.0037 ± 0.0002 ^d^	0.0048 ± 0.0002 ^ab^	0.0051 ± 0.0001 ^a^
18	1-Pentanol-M	1266.3	767.56	1.2529	0.0164 ± 0.0006 ^d^	0.0184 ± 0.0002 ^c^	0.0242 ± 0.0003 ^a^	0.0181 ± 0.0001 ^c^	0.0220 ± 0.0001 ^b^	0.0225 ± 0.0003 ^b^
19	1-Pentanol-D	1266.3	767.56	1.51425	0.0041 ± 0.0002 ^d^	0.0046 ± 0.0001 ^c^	0.0071 ± 0.0002 ^b^	0.0047 ± 0.0001 ^c^	0.0073 ± 0.0001 ^b^	0.0078 ± 0.0001 ^a^
20	2-Pentylfuran	1241.9	729.77	1.25488	0.0019 ± 0.0001 ^c^	0.0018 ± 0.0000 ^c^	0.0013 ± 0.0000 ^d^	0.0023 ± 0.0001 ^b^	0.0023 ± 0.0000 ^b^	0.0025 ± 0.0002 ^a^
21	(E)-2-Hexenal	1232.9	716.36	1.18361	0.0029 ± 0.0001 ^b^	0.0033 ± 0.0003 ^a^	0.0035 ± 0.0001 ^a^	0.0034 ± 0.0001 ^a^	0.0034 ± 0.0002 ^a^	0.0035 ± 0.0002 ^a^
22	3-Methyl-1-butanol	1222.1	700.51	1.24102	0.0076 ± 0.0002 ^d^	0.0079 ± 0.0003 ^d^	0.0107 ± 0.0002 ^a^	0.0095 ± 0.0002 ^b^	0.0084 ± 0.0001 ^c^	0.0086 ± 0.0004 ^c^
23	3-Methyl-2-butenal	1217.1	693.20	1.09451	0.0038 ± 0.0002 ^d^	0.0050 ± 0.0002 ^c^	0.0030 ± 0.0001 ^e^	0.0039 ± 0.0000 ^d^	0.0063 ± 0.0002 ^a^	0.0055 ± 0.0001 ^b^
24	Heptanal-M	1198	666.38	1.32814	0.0204 ± 0.0008 ^d^	0.0208 ± 0.0004 ^d^	0.0277 ± 0.0006 ^a^	0.0212 ± 0.0001 ^cd^	0.0224 ± 0.0003 ^b^	0.0217 ± 0.0002 ^bc^
25	Heptanal-D	1196.2	663.94	1.69838	0.0130 ± 0.0003 ^d^	0.0116 ± 0.0005 ^e^	0.0206 ± 0.0011 ^a^	0.0118 ± 0.0003 ^e^	0.0152 ± 0.0002 ^b^	0.0142 ± 0.0002 ^c^
26	2-Heptanone	1189.1	651.75	1.27072	0.0019 ± 0.0001 ^d^	0.0018 ± 0.0000 ^e^	0.0024 ± 0.0000 ^b^	0.0029 ± 0.0001 ^a^	0.0022 ± 0.0001 ^c^	0.0019 ± 0.0001 ^de^
27	1-Butanol-M	1163.8	599.34	1.18757	0.0270 ± 0.0004 ^d^	0.0279 ± 0.0008 ^c^	0.0345 ± 0.0001 ^a^	0.0321 ± 0.0001 ^b^	0.0285 ± 0.0002 ^c^	0.0278 ± 0.0004 ^c^
28	1-Butanol-D	1163.8	599.34	1.37764	0.0173 ± 0.0001 ^d^	0.0171 ± 0.0002 ^d^	0.0248 ± 0.0001 ^a^	0.0245 ± 0.0005 ^a^	0.0203 ± 0.0001 ^b^	0.0194 ± 0.0003 ^c^
29	(E)-2-Pentenal-M	1149.7	571.92	1.11043	0.0073 ± 0.0000 ^c^	0.0077 ± 0.0003 ^b^	0.0077 ± 0.0001 ^b^	0.0080 ± 0.0001 ^b^	0.0088 ± 0.0002 ^a^	0.0086 ± 0.0003 ^a^
30	(E)-2-Pentenal-D	1149.7	571.92	1.36044	0.0025 ± 0.0000 ^c^	0.0027 ± 0.0000 ^bc^	0.0018 ± 0.0001 ^d^	0.0027 ± 0.0002 ^b^	0.0034 ± 0.0001 ^a^	0.0035 ± 0.0002 ^a^
31	p-Xylene	1147	566.75	1.07323	0.0035 ± 0.0001 ^b^	0.0036 ± 0.0001 ^a^	0.0030 ± 0.0000 ^c^	0.0036 ± 0.0000 ^a^	0.0027 ± 0.0001 ^d^	0.0027 ± 0.0000 ^d^
32	Isoamyl acetate	1140.9	555.53	1.29049	0.0015 ± 0.0000 ^d^	0.0018 ± 0.0001 ^c^	0.0005 ± 0.0000 ^e^	0.0016 ± 0.0001 ^d^	0.0034 ± 0.0001 ^a^	0.0031 ± 0.0002 ^b^
33	2-Methyl-1-propanol-M	1111.5	503.76	1.16996	0.0118 ± 0.0001 ^e^	0.0127 ± 0.0001 ^d^	0.0158 ± 0.0001 ^a^	0.0134 ± 0.0002 ^c^	0.0127 ± 0.0001 ^d^	0.0139 ± 0.0003 ^b^
34	2-Methyl-1-propanol-D	1110.9	502.90	1.36192	0.0038 ± 0.0001 ^b^	0.0038 ± 0.0002 ^b^	0.0045 ± 0.0000 ^a^	0.0038 ± 0.0001 ^b^	0.0036 ± 0.0001 ^b^	0.0045 ± 0.0002 ^a^
35	Hexanal-M	1101	486.51	1.26371	0.0264 ± 0.0008 ^b^	0.0289 ± 0.0004 ^a^	0.0289 ± 0.0006 ^a^	0.0294 ± 0.0002 ^a^	0.0291 ± 0.0009 ^a^	0.0284 ± 0.0008 ^a^
36	Hexanal-D	1101.5	487.37	1.56877	0.0013 ± 0.0008 ^bc^	0.0019 ± 0.0011 ^b^	0.0010 ± 0.0006 ^c^	0.0008 ± 0.0004 ^b^	0.0039 ± 0.0023 ^a^	0.0037 ± 0.0021 ^a^
37	1-Penten-3-ol	1177.9	628.01	0.94227	0.0068 ± 0.0001 ^c^	0.0063 ± 0.0001 ^e^	0.0080 ± 0.0001 ^b^	0.0069 ± 0.0001 ^c^	0.0066 ± 0.0001 ^d^	0.0083 ± 0.0001 ^a^
38	Dimethyl disulfide	1084.2	462.35	1.13722	0.0016 ± 0.0000 ^c^	0.0020 ± 0.0001 ^a^	0.0009 ± 0.0001 ^e^	0.0019 ± 0.0001 ^b^	0.0014 ± 0.0000 ^d^	0.0013 ± 0.0001 ^d^
39	1-Propanol-M	1056	425.25	1.11341	0.0155 ± 0.0001 ^e^	0.0158 ± 0.0002 ^d^	0.0180 ± 0.0002 ^a^	0.0164 ± 0.0002 ^c^	0.0154 ± 0.0002 ^e^	0.0172 ± 0.0002 ^b^
40	1-Propanol-D	1056	425.25	1.24883	0.0082 ± 0.0001 ^d^	0.0083 ± 0.0000 ^d^	0.0100 ± 0.0001 ^b^	0.0086 ± 0.0001 ^d^	0.0094 ± 0.0003 ^c^	0.0111 ± 0.0004 ^a^
41	(E)-2-Butenal	1042.4	408.41	1.07797	0.0028 ± 0.0001 ^a^	0.0028 ± 0.0002 ^a^	0.0023 ± 0.0001 ^c^	0.0029 ± 0.0001 ^a^	0.0024 ± 0.0002 ^bc^	0.0025 ± 0.0000 ^b^
42	1-Penten-3-one	1014.2	375.677	1.08396	0.0019 ± 0.0002 ^d^	0.0029 ± 0.0000 ^a^	0.0017 ± 0.0000 ^e^	0.0027 ± 0.0001 ^b^	0.0021 ± 0.0001 ^c^	0.0020 ± 0.0000 ^cd^
43	Propyl acetate-M	1017.8	379.67	1.15094	0.0046 ± 0.0004 ^c^	0.0055 ± 0.0002 ^b^	0.0026 ± 0.0001 ^d^	0.0054 ± 0.0001 ^b^	0.0082 ± 0.0002 ^a^	0.0082 ± 0.0002 ^a^
44	Propyl acetate-D	1017.4	379.221	1.46822	0.0036 ± 0.0002 ^b^	0.0036 ± 0.0002 ^b^	0.0009 ± 0.0000 ^c^	0.0030 ± 0.0002 ^b^	0.0089 ± 0.0005 ^a^	0.0084 ± 0.0007 ^a^
45	Pentanal-M	1001	361.265	1.18176	0.0154 ± 0.0006 ^d^	0.0176 ± 0.0007 ^b^	0.0184 ± 0.0001 ^a^	0.0176 ± 0.0001 ^b^	0.0163 ± 0.0002 ^c^	0.0165 ± 0.0002 ^c^
46	Pentanal-D	1001.9	362.163	1.42371	0.0224 ± 0.0004 ^d^	0.0274 ± 0.0008 ^b^	0.0218 ± 0.0006 ^d^	0.0242 ± 0.0002 ^c^	0.0276 ± 0.0009 ^b^	0.0293 ± 0.0017 ^a^
47	Cyclohexanone	1300.3	823.935	1.1666	0.0019 ± 0.0000 ^a^	0.0015 ± 0.0001 ^b^	0.0015 ± 0.0000 ^b^	0.0016 ± 0.0001 ^b^	0.0013 ± 0.0001 ^c^	0.0015 ± 0.0001 ^b^
48	Ethanol-M	946.6	318.44	1.04367	0.0468 ± 0.0018 ^b^	0.0440 ± 0.0007 ^c^	0.0499 ± 0.0003 ^a^	0.0407 ± 0.0005 ^d^	0.0370 ± 0.0000 ^e^	0.0363 ± 0.0006 ^e^
49	Ethanol-D	946	317.955	1.12702	0.1008 ± 0.0030 ^a^	0.0925 ± 0.0017 ^b^	0.1017 ± 0.0009 ^a^	0.0848 ± 0.0015 ^c^	0.0799 ± 0.0013 ^d^	0.0747 ± 0.0011 ^e^
50	Ethyl propanoate	969	334.926	1.47248	0.0010 ± 0.0000 ^b^	0.0009 ± 0.0001 ^b^	0.0008 ± 0.0000 ^c^	0.0012 ± 0.0000 ^a^	0.0013 ± 0.0000 ^a^	0.0012 ± 0.0001 ^a^
51	2-Propanol	936.4	311.167	1.08715	0.0026 ± 0.0001 ^d^	0.0034 ± 0.0001 ^b^	0.0045 ± 0.0002 ^a^	0.0025 ± 0.0000 ^d^	0.0027 ± 0.0001 ^cd^	0.0029 ± 0.0001 ^c^
52	3-Methylbutanal	928	305.348	1.40363	0.0135 ± 0.0006 ^b^	0.0100 ± 0.0004 ^c^	0.0093 ± 0.0007 ^cd^	0.0151 ± 0.0010 ^a^	0.0087 ± 0.0002 ^d^	0.0085 ± 0.0001 ^d^
53	Tert-Butanol	934.3	309.712	1.32512	0.0129 ± 0.0008 ^a^	0.0109 ± 0.0005 ^bc^	0.0113 ± 0.0002 ^b^	0.0114 ± 0.0007 ^b^	0.0101 ± 0.0002 ^c^	0.0105 ± 0.0008 ^bc^
54	Ethyl Acetate	905.6	290.317	1.32632	0.0051 ± 0.0002 ^c^	0.0044 ± 0.0000 ^e^	0.0081 ± 0.0000 ^a^	0.0067 ± 0.0002 ^b^	0.0048 ± 0.0001 ^d^	0.0043 ± 0.0001 ^e^
55	2-Butanone	915.8	297.106	1.25022	0.0075 ± 0.0003 ^b^	0.0073 ± 0.0005 ^b^	0.0069 ± 0.0001 ^b^	0.0071 ± 0.0006 ^b^	0.0093 ± 0.0002 ^a^	0.0088 ± 0.0003 ^a^
56	Diethyl acetal	910.7	293.711	1.02917	0.0314 ± 0.0001 ^bc^	0.0334 ± 0.0015 ^ab^	0.0362 ± 0.0004 ^a^	0.0336 ± 0.0003 ^ab^	0.0298 ± 0.0022 ^c^	0.0314 ± 0.0035 ^bc^
57	Butanal-M	890.5	280.62	1.11252	0.0063 ± 0.0004 ^c^	0.0065 ± 0.0002 ^c^	0.0086 ± 0.0000 ^a^	0.0078 ± 0.0002 ^b^	0.0067 ± 0.0002 ^c^	0.0064 ± 0.0003 ^c^
58	Butanal-D	889	279.65	1.28405	0.0057 ± 0.0000 ^bc^	0.0044 ± 0.0002 ^d^	0.0061 ± 0.0001 ^b^	0.0066 ± 0.0002 ^a^	0.0052 ± 0.0002 ^c^	0.0047 ± 0.0005 ^d^
59	Methacrolein	895.8	284.014	1.22365	0.0069 ± 0.0002 ^b^	0.0084 ± 0.0001 ^a^	0.0023 ± 0.0001 ^d^	0.0061 ± 0.0001 ^c^	0.0084 ± 0.0005 ^a^	0.0081 ± 0.0008 ^a^
60	Acrolein	867.7	266.558	1.0642	0.0084 ± 0.0003 ^c^	0.0092 ± 0.0002 ^b^	0.0037 ± 0.0001 ^e^	0.0110 ± 0.0009 ^a^	0.0066 ± 0.0001 ^d^	0.0065 ± 0.0002 ^d^
61	Acetone	837.6	249.102	1.11614	0.0913 ± 0.0019 ^c^	0.0949 ± 0.0006 ^b^	0.0812 ± 0.0003 ^d^	0.0910 ± 0.0006 ^c^	0.1015 ± 0.0008 ^a^	0.1033 ± 0.0013 ^a^
62	Propanal	819.1	238.92	1.14634	0.0211 ± 0.0005 ^bc^	0.0215 ± 0.0002 ^b^	0.0200 ± 0.0004 ^d^	0.0204 ± 0.0002 ^cd^	0.0214 ± 0.0004 ^b^	0.0229 ± 0.0007 ^a^
63	Ethyl formate	829.8	244.738	1.20795	0.0048 ± 0.0002 ^a^	0.0038 ± 0.0001 ^c^	0.0044 ± 0.0001 ^b^	0.0048 ± 0.0001 ^a^	0.0033 ± 0.0001 ^d^	0.0033 ± 0.0000 ^d^
64	2-Methylpropanal	830.6	245.223	1.28163	0.0009 ± 0.0001 ^b^	0.0006 ± 0.0000 ^c^	0.0008 ± 0.0001 ^b^	0.0012 ± 0.0001 ^a^	0.0006 ± 0.0000 ^cd^	0.0005 ± 0.0001 ^d^
65	Dimethyl sulphide	797.9	227.768	0.96032	0.0162 ± 0.0004 ^a^	0.0111 ± 0.0003 ^d^	0.0071 ± 0.0002 ^e^	0.0154 ± 0.0005 ^b^	0.0109 ± 0.0002 ^d^	0.0119 ± 0.0003 ^c^
66	Acetaldehyde	764.5	211.282	0.97965	0.0200 ± 0.0025 ^b^	0.0246 ± 0.0057 ^ab^	0.0257 ± 0.0007 ^a^	0.0237 ± 0.0012 ^ab^	0.0225 ± 0.0017 ^ab^	0.0210 ± 0.0004 ^ab^
67	2-Pentanone	1000.4	360.625	1.3529	0.0068 ± 0.0001 ^d^	0.0075 ± 0.0003 ^bc^	0.0083 ± 0.0000 ^a^	0.0072 ± 0.0002 ^cd^	0.0077 ± 0.0002 ^b^	0.0072 ± 0.0002 ^c^
68	2-Butylfuran	1147.1	567.018	1.18523	0.0022 ± 0.0002 ^a^	0.0017 ± 0.0001 ^b^	0.0015 ± 0.0001 ^bc^	0.0013 ± 0.0000 ^c^	0.0022 ± 0.0001 ^a^	0.0013 ± 0.0000 ^c^
69	2,3-Pentanedione	1057.9	427.626	1.31919	0.0015 ± 0.0001 ^b^	0.0019 ± 0.0001 ^a^	0.0007 ± 0.0000 ^d^	0.0020 ± 0.0001 ^a^	0.0014 ± 0.0001 ^bc^	0.0012 ± 0.0002 ^c^
70	4-Methyl-2-pentanone	1027.8	391.09	1.1818	0.0021 ± 0.0001 ^c^	0.0026 ± 0.0004 ^b^	0.0017 ± 0.0000 ^d^	0.0016 ± 0.0001 ^d^	0.0031 ± 0.0001 ^a^	0.0030 ± 0.0001 ^a^
71	2-Butanol	1039.8	405.311	1.14473	0.0020 ± 0.0002 ^b^	0.0016 ± 0.0001 ^c^	0.0022 ± 0.0001 ^a^	0.0016 ± 0.0000 ^c^	0.0020 ± 0.0001 ^ab^	0.0021 ± 0.0001 ^ab^
72	Beta-Pinene	1136.2	546.908	1.21146	0.0007 ± 0.0001 ^f^	0.0008 ± 0.0001 ^e^	0.0018 ± 0.0001 ^a^	0.0011 ± 0.0000 ^d^	0.0014 ± 0.0000 ^b^	0.0012 ± 0.0000 ^c^
73	Ethyl 3-methylbutanoate	1087.3	466.525	1.25223	0.0007 ± 0.0000 ^c^	0.0007 ± 0.0001 ^c^	0.0011 ± 0.0000 ^a^	0.0009 ± 0.0000 ^b^	0.0006 ± 0.0000 ^d^	0.0005 ± 0.0000 ^d^
74	2,5-Dimethylfuran	949.6	320.601	1.3758	0.0004 ± 0.0000 ^c^	0.0005 ± 0.0000 ^c^	0.0002 ± 0.0000 ^d^	0.0009 ± 0.0000 ^b^	0.0023 ± 0.0001 ^a^	0.0024 ± 0.0002 ^a^
75	Isobutyl acetate	1030.4	394.181	1.23494	0.0016 ± 0.0000 ^c^	0.0017 ± 0.0000 ^b^	0.0009 ± 0.0001 ^d^	0.0016 ± 0.0000 ^c^	0.0026 ± 0.0001 ^a^	0.0026 ± 0.0001 ^a^
76	3-Methyl-2-pentanone	1036.7	401.601	1.47712	0.0004 ± 0.0000 ^b^	0.0003 ± 0.0000 ^bc^	0.0002 ± 0.0000 ^c^	0.0003 ± 0.0000 ^bc^	0.0009 ± 0.0000 ^a^	0.0009 ± 0.0002 ^a^
77	4-Methyl-3-penten-2-one	1127.9	532.068	1.11137	0.0010 ± 0.0001 ^a^	0.0008 ± 0.0000 ^b^	0.0008 ± 0.0001 ^b^	0.0008 ± 0.0000 ^b^	0.0010 ± 0.0001 ^a^	0.0010 ± 0.0000 ^a^
78	Myrcene	1176.1	624.198	1.20404	0.0010 ± 0.0000 ^b^	0.0009 ± 0.0000 ^c^	0.0008 ± 0.0000 ^c^	0.0008 ± 0.0001 ^c^	0.0015 ± 0.0001 ^a^	0.0011 ± 0.0001 ^b^
79	2-Methyl-2-pentenal	1173.7	619.252	1.51296	0.0005 ± 0.0000 ^b^	0.0005 ± 0.0000 ^b^	0.0004 ± 0.0000 ^b^	0.0004 ± 0.0000 ^b^	0.0016 ± 0.0001 ^a^	0.0016 ± 0.0003 ^a^
80	Ethyl crotonate	1178.4	629.145	1.17933	0.0014 ± 0.0001 ^e^	0.0016 ± 0.0001 ^d^	0.0012 ± 0.0000 ^f^	0.0018 ± 0.0001 ^c^	0.0026 ± 0.0001 ^a^	0.0020 ± 0.0000 ^b^
81	Butyl butanoate	1223	701.753	1.3399	0.0017 ± 0.0001 ^c^	0.0018 ± 0.0001 ^bc^	0.0008 ± 0.0000 ^d^	0.0016 ± 0.0001 ^c^	0.0024 ± 0.0002 ^a^	0.0019 ± 0.0000 ^b^
82	Ethyl hexanoate	1249.3	741.113	1.33342	0.0013 ± 0.0001 ^c^	0.0015 ± 0.0001 ^b^	0.0011 ± 0.0001 ^d^	0.0015 ± 0.0001 ^b^	0.0024 ± 0.0001 ^a^	0.0022 ± 0.0001 ^a^
83	2-Decenal	1758.5	2227.265	1.49379	0.0060 ± 0.0009 ^a^	0.0055 ± 0.0005 ^a^	0.0057 ± 0.0013 ^a^	0.0055 ± 0.0008 ^a^	0.0071 ± 0.0008 ^a^	0.0063 ± 0.0004 ^a^
84	Butanoic acid	1710.1	2005.275	1.16111	0.0033 ± 0.0001 ^c^	0.0032 ± 0.0001 ^c^	0.0040 ± 0.0001 ^b^	0.0034 ± 0.0003 ^c^	0.0033 ± 0.0001 ^c^	0.0046 ± 0.0003 ^a^
85	Isobutanoic acid	1628.5	1679.983	1.15864	0.0053 ± 0.0001 ^bc^	0.0043 ± 0.0006 ^d^	0.0058 ± 0.0005 ^b^	0.0051 ± 0.0003 ^c^	0.0047 ± 0.0003 ^cd^	0.0066 ± 0.0002 ^a^
86	Acetoin	1299.4	822.47	1.05843	0.0151 ± 0.0017 ^a^	0.0104 ± 0.0009 ^b^	0.0103 ± 0.0004 ^b^	0.0103 ± 0.0002 ^b^	0.0079 ± 0.0003 ^c^	0.0091 ± 0.0002 ^bc^
87	1-Octen-3-one	1310.7	842.906	1.26942	0.0013 ± 0.0001 ^bc^	0.0013 ± 0.0001 ^bc^	0.0012 ± 0.0001 ^c^	0.0014 ± 0.0000 ^b^	0.0020 ± 0.0002 ^a^	0.0019 ± 0.0000 ^a^
88	(Z)-Hex-3-enol	1416.7	1060.894	1.26747	0.0012 ± 0.0000 ^c^	0.0012 ± 0.0001 ^bc^	0.0010 ± 0.0001 ^d^	0.0012 ± 0.0001 ^c^	0.0014 ± 0.0001 ^a^	0.0014 ± 0.0001 ^ab^
89	2-Heptanol	1348.5	914.92	1.37883	0.0011 ± 0.0001 ^b^	0.0011 ± 0.0001 ^b^	0.0005 ± 0.0002 ^c^	0.0010 ± 0.0001 ^b^	0.0015 ± 0.0001 ^a^	0.0015 ± 0.0001 ^a^
90	Ethyl heptanoate	1339.1	896.43	1.40618	0.0008 ± 0.0001 ^c^	0.0010 ± 0.0001 ^ab^	0.0006 ± 0.0001 ^d^	0.0009 ± 0.0000 ^bc^	0.0010 ± 0.0000 ^a^	0.0009 ± 0.0000 ^abc^
91	Styrene	1269.3	772.356	1.44107	0.0017 ± 0.0000 ^d^	0.0018 ± 0.0000 ^d^	0.0020 ± 0.0001 ^c^	0.0026 ± 0.0001 ^a^	0.0026 ± 0.0001 ^a^	0.0022 ± 0.0001 ^b^
92	2-Acetyl-1-pyrroline	1344.5	907.058	1.13317	0.0009 ± 0.0002 ^a^	0.0009 ± 0.0001 ^a^	0.0005 ± 0.0001 ^b^	0.0008 ± 0.0001 ^a^	0.0005 ± 0.0001 ^b^	0.0005 ± 0.0000 ^b^
93	**1**	1054	422.658	0.94674	0.0053 ± 0.0002 ^cd^	0.0055 ± 0.0002 ^c^	0.0051 ± 0.0000 ^d^	0.0046 ± 0.0001 ^e^	0.0077 ± 0.0002 ^a^	0.0065 ± 0.0002 ^b^
94	**2**	1031.1	394.929	1.08516	0.0036 ± 0.0011 ^a^	0.0031 ± 0.0017 ^a^	0.0033 ± 0.0012 ^a^	0.0025 ± 0.0015 ^a^	0.0030 ± 0.0032 ^a^	0.0025 ± 0.0028 ^a^
95	**3**	971.6	336.866	1.4145	0.0019 ± 0.0001 ^bc^	0.0014 ± 0.0001 ^d^	0.0021 ± 0.0001 ^a^	0.0020 ± 0.0001 ^b^	0.0021 ± 0.0000 ^a^	0.0017 ± 0.0000 ^c^
96	**4**	950	320.865	1.24056	0.0126 ± 0.0002 ^d^	0.0123 ± 0.0001 ^d^	0.0116 ± 0.0001 ^e^	0.0129 ± 0.0003 ^c^	0.0151 ± 0.0001 ^a^	0.0144 ± 0.0000 ^b^
97	**5**	851.2	256.86	1.15842	0.0019 ± 0.0001 ^b^	0.0016 ± 0.0001 ^c^	0.0025 ± 0.0001 ^a^	0.0015 ± 0.0001 ^c^	0.0010 ± 0.0001 ^d^	0.0011 ± 0.0001 ^d^
98	**6**	790.3	223.889	1.09199	0.0223 ± 0.0006 ^a^	0.0175 ± 0.0005 ^c^	0.0099 ± 0.0001 ^f^	0.0188 ± 0.0003 ^b^	0.0134 ± 0.0004 ^d^	0.0116 ± 0.0002 ^e^
99	**7**	1015.1	376.626	1.39759	0.0020 ± 0.0001 ^d^	0.0025 ± 0.0001 ^c^	0.0005 ± 0.0000 ^e^	0.0020 ± 0.0001 ^d^	0.0031 ± 0.0002 ^a^	0.0029 ± 0.0002 ^b^
100	**8**	1056.7	426.084	1.44228	0.0006 ± 0.0000 ^b^	0.0009 ± 0.0001 ^a^	0.0003 ± 0.0000 ^c^	0.0009 ± 0.0001 ^a^	0.0006 ± 0.0000 ^b^	0.0006 ± 0.0001 ^b^
101	**9**	1029.9	393.563	1.50307	0.0002 ± 0.0000 ^c^	0.0004 ± 0.0000 ^b^	0.0001 ± 0.0000 ^d^	0.0002 ± 0.0000 ^c^	0.0007 ± 0.0000 ^a^	0.0007 ± 0.0001 ^a^
102	**10**	1135.2	545.053	1.09036	0.0010 ± 0.0001 ^a^	0.0009 ± 0.0002 ^a^	0.0005 ± 0.0000 ^b^	0.0010 ± 0.0000 ^a^	0.0009 ± 0.0000 ^a^	0.0010 ± 0.0001 ^a^
103	**11**	1395.5	1013.209	1.27333	0.0009 ± 0.0000 ^b^	0.0010 ± 0.0001 ^b^	0.0008 ± 0.0001 ^c^	0.0009 ± 0.0001 ^b^	0.0013 ± 0.0001 ^a^	0.0010 ± 0.0000 ^b^

Note: D—dimer; M—monomer; different lowercase letters in the same row indicate significant differences (*p* < 0.05). The numbers in the compound represent unknown compounds.

**Table 2:** Aroma genotypes of aromatic rice Diantun502 and its improved lines.

Abbreviation	Plant Height	Effective Spikelet	Waxy	Aromatic	Genotype
DT502	101.0	8.3	s	a	*badh2/badh2*
DG163	137.7	10.4	s	a	*badh2/badh2*
DG1839	108.4	9.2	s	a	*badh2/badh2*
DG1946	109.2	11.0	s	a	*badh2/badh2*
DG1938	129.4	9.7	s	n	*badh2/badh2*
DG2029	118.6	10.1	g	a	*badh2/badh2*
DG2030	122.2	8.6	g	a	*badh2/badh2*

Note: a: aromatic trait; n: nonaromatic trait; s: sticky rice; g: glutinous rice.

**Table 3:** Special primers used for aromatic gene badh2 locus amplification in rice.

Marker	Primer Sequence (5′-3′)	Source of Primer
ESP	P1:TTGTTTGGAGCTTGCTGATG	Bradbury et al. (2005) [45]
IFAP	P2:CATAGGAGCAGCTGAAATATATACC	
INSP	P3:CTGGTAAAAAGATTATGGCTTCA	
EAP	P4:AGTGCTTTACAAAGTCCCGC	

## Data Availability

The data presented in this study are available on request from the corresponding author.
